# Stepped-Care Web-Based Parent Support Following Congenital Heart Disease: Protocol for a Randomized Controlled Trial

**DOI:** 10.2196/64216

**Published:** 2024-10-04

**Authors:** Marin Taylor, Bianca Christina Bondi, Brendan F Andrade, Stephanie H Au-Young, Vann Chau, Ashley Danguecan, Naddley Désiré, Ting Guo, Dragana Ostojic-Aitkens, Shari Wade, Steven Miller, Tricia Samantha Williams

**Affiliations:** 1 The Department of Psychology The Hospital for Sick Children Toronto, ON Canada; 2 Margaret and Wallace McCain Centre for Child, Youth & Family Mental Health Centre for Addiction and Mental Health Toronto, ON Canada; 3 Campbell Family Mental Health Research Institute Centre for Addiction and Mental Health Toronto, ON Canada; 4 Department of Neurology The Hospital for Sick Children Toronto, ON Canada; 5 Division of Rehabilitation Medicine Cincinnati Children's Hospital Medical Center Cincinnati, OH United States; 6 Department of Pediatrics University of Cincinnati College of Medicine Cincinnati, OH United States; 7 Pediatric Medicine BC Children's Hospital Vancouver, BC Canada; 8 Department of Psychiatry University of Toronto Toronto, ON Canada

**Keywords:** congenital heart disease, neurodevelopmental outcomes, web-based mental health care, stepped care, positive parenting, family well-being, mental health, I-InTERACT-North

## Abstract

**Background:**

Early neurodevelopmental risks, compounded with traumatic medical experiences, contribute to emotional and behavioral challenges in as many as 1 in 2 children with congenital heart disease (CHD). Parents report a strong need for supports; yet, there remains a lack of accessible, evidence-based behavioral interventions available for children with CHD and their families. I-InTERACT-North is a web-based stepped-care mental health program designed to support family well-being and reduce behavioral concerns through positive parenting for children with early medical complexity. In previous pilot studies, the program was effective in increasing positive parenting skills and decreasing child behavior problems, with high parent-reported acceptability. This paper presents the protocol for the first randomized study of stepped-care parent support for families of children with CHD.

**Objective:**

This study will involve a single-site, 2-arm, single-blind randomized controlled trial to evaluate (1) the feasibility and acceptability of a web-based stepped-care parent support program (I-InTERACT-North) and (2) the effectiveness of the program in enhancing positive parenting skills and reducing behavioral concerns among families of children with CHD.

**Methods:**

Families will be randomized (1:1) to either receive treatment or continue with care as usual for 12 months. Randomization will be stratified by child’s sex assigned at birth and baseline parent-reported child behavior intensity. Primary outcomes include positive parenting skills and child behavior at baseline, 3 months, 6 months, and 12 months. Secondary outcomes include parental mental health, quality of life, service usage, and feasibility including program reach and adherence. A sample size of 244 families will provide >95% power to detect an effect size of *d*=0.64. Based on attrition data from pilot studies, a target of 382 families will be enrolled. Parent reports of acceptability, adoption, and suggested adaptability of the program will be examined using cross-case thematic analyses. Primary efficacy analysis will be conducted using an intent-to-treat approach. Generalized estimating equations will be used to examine changes in positive parenting. Child behavior, quality of life, and parent mental health will be tested with repeated-measures analyses. Additional sensitivity and replication analyses will also be carried out.

**Results:**

Recruitment began in February 2024, and recruitment and follow-up will continue until January 2029. We anticipate results in late 2029.

**Conclusions:**

This study aims to test the effectiveness of I-InTERACT-North web-based stepped-care parent support in improving positive parenting skills and reducing child behavior problems in families of children with CHD compared with a care as usual control group. Results will inform future clinical implementation and expansion of this program among families of children with early medical conditions.

**Trial Registration:**

ClinicalTrials.gov NCT06075251; https://clinicaltrials.gov/study/NCT06075251

**International Registered Report Identifier (IRRID):**

DERR1-10.2196/64216

## Introduction

Congenital heart disease (CHD) is the world’s leading birth defect, impacting 1% of Canadian children [1,2]. Advances in medical care have significantly enhanced the survival rate post CHD diagnosis; however, children with CHD continue to face greater and persistent challenges in learning, behavior, and socioemotional development than their healthy-born peers [3]. Adverse neurodevelopmental outcomes are influenced by evolving genetic, cardiovascular, and psychosocial risk factors in early childhood [4]. These early risks, compounded with the emotional toll of traumatic medical experiences for children and parents (the word “parent” is used here and throughout this paper to denote the primary caregivers; this may include biological parents, adoptive parents, foster parents, grandparents, family members, or other individuals responsible for childcare), can contribute to the emergence of emotional and behavior problems (eg, noncompliance, acting out) in as many as 1 in 2 children with CHD [5-11]. Yet, a gap remains in the availability of accessible, evidence-based treatments to support the social, emotional, and behavioral needs of children and families affected by CHD [12-14].

When considering factors affecting children’s development, parenting and parent-child relationships emerge as potential modifiable determinants of health. Indeed, positive parenting, which includes parental flexibility, responsiveness, constructive parenting, and emotional warmth [15], is a well-established universal protective factor promoting optimal child emotional and behavioral outcomes [16,17]. Several widely used mental health interventions addressing childhood emotional and behavioral problems target positive parenting skills, aiming to strengthen parent-child interactions to improve child behavior [18-20]. Examples include the Incredible Years Parenting Program [21] and the Positive Parenting Program (Triple P [22]). These programs target children identified as at-risk based on socioeconomic factors; however, in recent years, investigators have considered the potential for positive parenting to optimize outcomes among medically at-risk children [23,24]. In our work among children with CHD and their families, parents prioritized mental health support for their children and themselves to empower their parenting skills and confidence. Parents also noted the burdens associated with additional in-person mental health programming, highlighting the need for more accessible web-based services [14].

In response to these identified needs, our team at The Hospital for Sick Children developed the I-InTERACT-North program with and for families of children with CHD by adapting components from the parenting program I-InTERACT (Internet-based Interacting Together Everyday Recovery from Traumatic Brain Injury [25]). I-InTERACT was developed at the Cincinnati Children’s Hospital Medical Centre by Wade and colleagues [25] and uses in vivo coaching to improve positive parenting skills in a telehealth format. The original I-InTERACT program was shown to improve positive parenting skills and child behavior following pediatric traumatic brain injury (TBI [25,26]). While children with CHD share some underlying vulnerabilities with children with TBI, parents of children with CHD have reflected how their prenatal and neonatal experiences were unique from parents of children with TBI within an initial needs assessment [14]. Specifically, parents described stress beginning prior to or at birth, followed by significant fears and uncertainty during their child’s early medical and developmental journey. With this input, the I-InTERACT-North program caters specifically to families of children with congenital and neonatal conditions, including CHD, by providing new information regarding the unique neurodevelopmental and psychosocial impact of these conditions [27]. Likewise, program strategies are tailored to family-specific behavioral concerns, including psychoeducation, coaching, and problem-solving, to address their individual child’s needs in the context of their medical and treatment history. Following an initial pilot, which found I-InTERACT-North to be feasible, acceptable, and effective in increasing positive parenting skills and decreasing child behavior problems [28], our group further adapted the program to a novel stepped-care model, as informed by patient-orientated approaches, in which families can access needs-matched incremental supports (ie, an introduction session, an abbreviated program, and a full program [29]). Pilot data from the stepped-care program has demonstrated high acceptability, with strong parent-reported accessibility and adaptability of care to their family’s needs. Likewise, pilot data demonstrated the program’s success in improving positive parenting skills (*P*<.001; η^2^=0.850) and child behavior problems (*P*=.001; *d*=0.390). Without sacrificing efficacy, stepped-care I-InTERACT-North delivery also improves efficiency and contributes to precision mental health by offering scalable services that are adaptable to the needs of families.

To date, efficacy and feasibility of I-InTERACT-North have not been systematically investigated in children with CHD exclusively using a randomized controlled trial (RCT) design. This study aims to test the effectiveness of I-InTERACT-North in improving positive parenting skills and reducing child behavior problems in families of children with CHD compared with care as usual (CAU). This manuscript provides rationale for and information regarding the stepped-care parent support program and describes how intervention effectiveness will be evaluated in families of children with CHD. This study protocol manuscript was developed and submitted before data collection and analyses were completed.

## Methods

### Study Design

The proposed hybrid type 1 study is a single-site, 2-arm, single-blind RCT designed to simultaneously assess (1) feasibility and acceptability of an RCT of stepped-care web-based parent support (ie, adherence, fidelity, costs, and acceptability) and (2) efficacy of the stepped-care I-InTERACT-North program in improving positive parenting skills and child behavior among families of children with CHD.

Hypothesis 1: based on our pilot data, we hypothesize that the stepped-care program will demonstrate program feasibility (ie, adherence and fidelity, >70%) and high acceptability (ie, based on postintervention program ratings, thematic analysis of feedback, >90%) across CHD families.

Hypothesis 2: we hypothesize that positive parenting skills and child behavior will improve at the end of the treatment among participants in the treatment arm compared to CAU and remain improved compared with CAU at 12 months. We consider positive parenting skills as the primary proximal outcome and child behavior (co–primary outcome) as mediated by improvements in parenting. We also consider secondary outcome changes in parent mental health and family quality of life.

**Figure 1:**
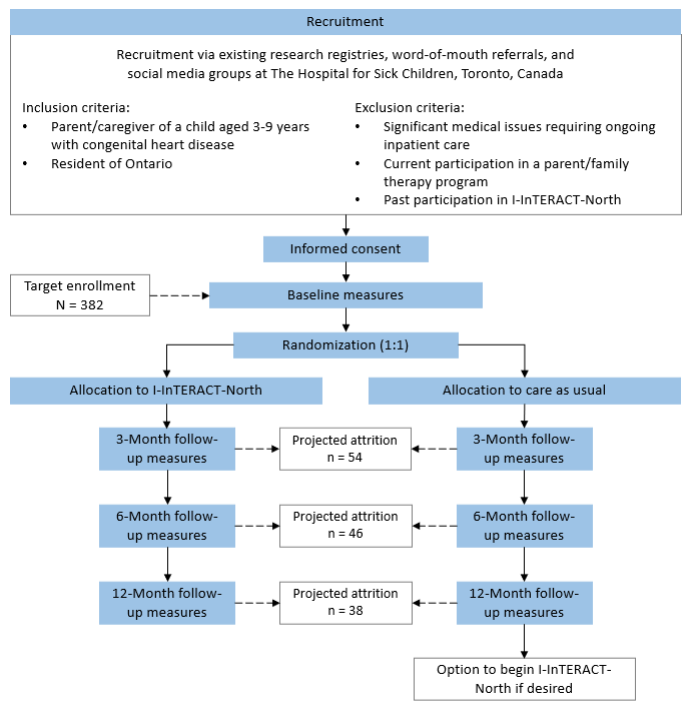
Flowchart of planned study recruitment, retention, and procedures. I-InTERACT: Internet-based Interacting Together Everyday Recovery from Traumatic Brain Injury.

After completion of baseline measures, families will be randomized to receive CAU or active I-InTERACT-North stepped-care treatment. Randomization will be stratified by sex assigned at birth (male, female) and baseline child behavior severity (Eyberg Child Behavior Inventory [ECBI] intensity *t* score above or below cutoff of 60) and executed by a computerized randomization system on REDCap (Research Electronic Data Capture; Vanderbilt University) [30]. Families in both conditions will complete follow-up measures at 3 months, 6 months, and 12 months postbaseline (Figure 1). Families randomized to CAU will receive no direct parent treatment other than clinical care provided in cardiac follow-up (ie, child assessment and consultation), which will be documented accordingly. The CAU families will be offered I-InTERACT-North after completion of their 12-month follow-up measurements. Participants cannot be blinded to their condition, but they will be blinded to primary outcomes and hypotheses. Independent study staff responsible for coding the primary outcomes (ie, positive parenting skills) will be blind to participant allocation, outcomes, and baseline or follow-up status.

### Ethical Considerations

This study was approved by the research ethics board at The Hospital for Sick Children (1000080752). Given that the study participants are parents, all will provide written, informed consent. It is specified that participants can withdraw their consent at any given time in the study upon request. All patient data will be deidentified and stored in secure locations. Families are compensated with a CAD $25 (US $18.47) gift card at each timepoint (ie, baseline, 3 months, 6 months, and 12 months).

### Participants

Participants will be recruited from The Hospital for Sick Children in Toronto, Ontario, which is the largest cardiac care center in Canada. Eligible participants must be parents of a child aged 3-9 years (at time of enrollment) with CHD followed at The Hospital for Sick Children. The family must reside in the province of Ontario given professional jurisdiction pertaining to psychological care provision. Exclusion criteria include significant medical issues requiring inpatient care, current participation in a similar family or parent therapy program (eg, Incredible Years Parenting Program [21] and Positive Parenting Program [Triple P] [22]), or previous participation in I-InTERACT-North pilot studies. There are no exclusions based on medical or mental health diagnosis; however, any patients undergoing significant issues requiring inpatient care will be deferred and reapproached at a more appropriate time. To overcome access inclusion criteria, we will provide families in need with tablets based on our Canadian pilot (5%) and Canadian web-based coverage status. Interpreter services are also available for recruitment, informed consent, and I-InTERACT-North treatment sessions.

### Recruitment

Participants will be recruited through three channels: (1) existing cardiology and neurology research registries at The Hospital for Sick Children (REB #1000051521 and #1000052378) comprising families of children with CHD who have consented to future contact regarding research studies, (2) word-of-mouth recruitment by coinvestigators and collaborators providing clinical care to eligible families, and (3) social media recruitment via Facebook groups moderated by parents for CHD caregivers at The Hospital for Sick Children.

### Intervention

**Figure 2:**
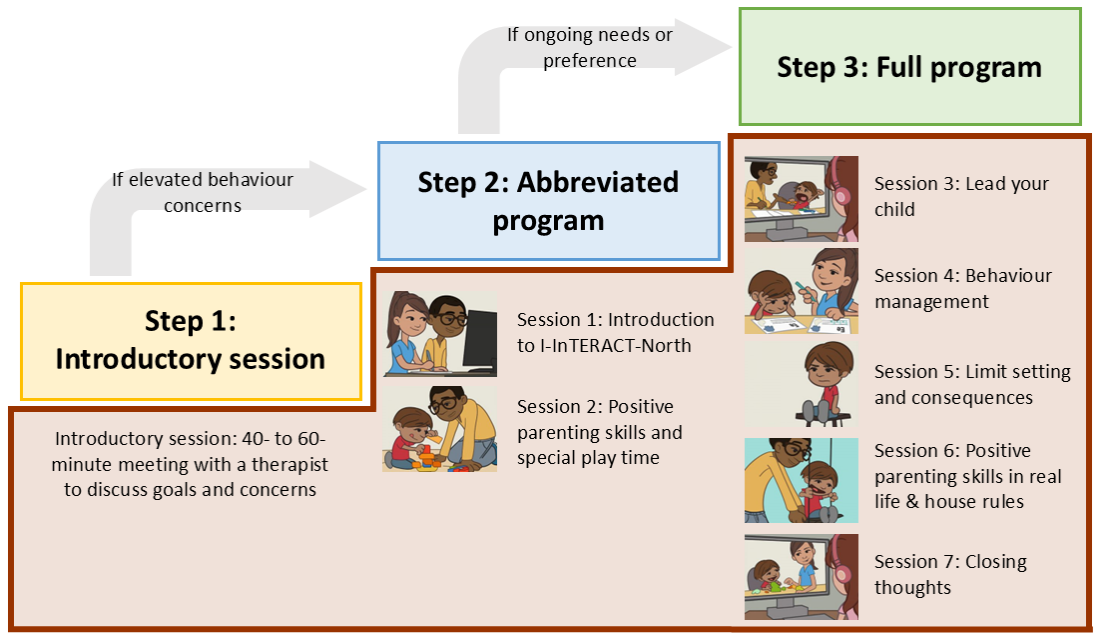
Stepped-care model of I-InTERACT-North. I-InTERACT: Internet-based Interacting Together Everyday Recovery from Traumatic Brain Injury.

**Figure 3:**
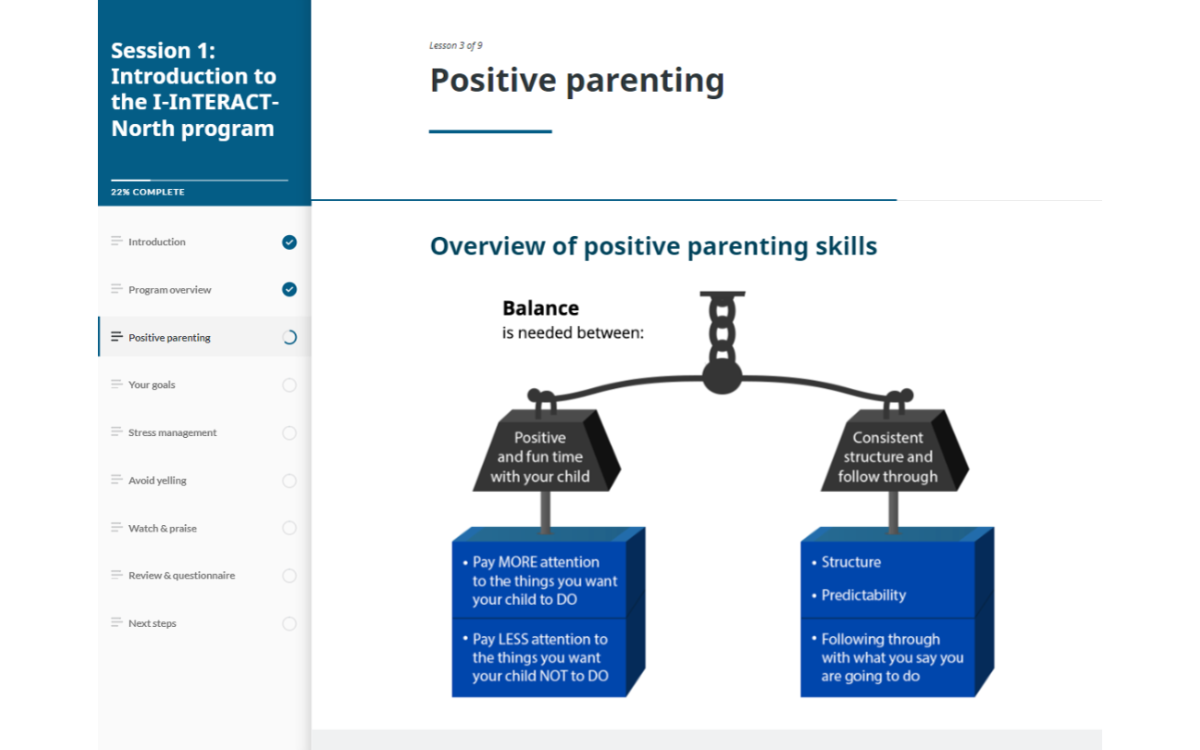
I-InTERACT-North web-based psychoeducational module. I-InTERACT: Internet-based Interacting Together Everyday Recovery from Traumatic Brain Injury.

I-InTERACT-North is a stepped-care web-based parenting program consisting of 3 tiered steps of increasing therapeutic intensity (Figure 2). In step 1, participants are matched with a therapist for an introductory appointment to review their concerns and goals for the program. Steps 2 and 3 combine web-based therapy sessions with web-based psychoeducational modules that introduce positive parenting skills (eg, labeled praise, reflective responses; Figure 3) as well as child behavior management strategies. Each web-based module concludes with a brief quiz to assess engagement and understanding. Parents complete the web-based modules asynchronously before their web-based therapy appointment, where they review the strategies and engage in active coaching with their therapist while their child is present. Step 2 includes the first 2 therapy sessions and step 3 consists of an additional 5 sessions. Depending on the number of steps completed, as well as scheduling of sessions, I-InTERACT-North may last anywhere from 1 day (ie, 1 single-hour session) up to 7 weeks (for weekly sessions) or 14 weeks (for biweekly sessions).

A collaborative approach to treatment planning (ie, step-up or step-down as outlined by Kennedy et al [31]) is used with families at the end of steps 1 and 2 to decide whether they will proceed with treatment. Step-up indicators include low positive parenting skills as coded from observed parent-child interactions, high child behavior severity, and high in-session ratings of the family’s “top problems” [31,32].

Each session follows a manualized therapy protocol to optimize therapist fidelity. All sessions are recorded (with written consent from the participant) and reviewed in supervision. Therapy sessions are delivered via Zoom (Zoom Video Communications, Inc) [33]. All I-InTERACT-North therapists possess masters-level (or higher) education in clinical psychology and come from diverse cultural and language backgrounds. All therapists undergo extensive training and ongoing supervision to ensure program fidelity; this includes a 2-day synchronous training course, 3-6 months of case shadowing, and regular individual and group supervision by a licensed psychologist upon commencing independent program delivery.

### Measures

#### Overview

Data collected in this RCT include video recordings of parent-child interactions, medical chart extractions, and parent-report questionnaires. All participant-reported measures are completed via REDCap [30] at baseline, 3 months, 6 months, and 12 months (Table 1).

**Table 1:** Baseline and outcome measures by timepoint.

Measure	Timepoint			
	Baseline	3 Months (± 3 weeks)	6 Months (± 3 weeks)	12 Months (± 3 weeks)
Chart review	✓			
DPICS^a^	✓	✓	✓	✓
ECBI^b^	✓	✓	✓	✓
DASS^c^	✓	✓	✓	✓
EQ-5D-5L	✓	✓	✓	✓
CSRI^d^	✓	✓	✓	✓
Parent Acceptability Questionnaire^e^		✓		

^a^DPICS: Dyadic Parent-Child Interaction Coding System.

^b^ECBI: Eyberg Child Behavior Inventory.

^c^DASS: Depression and Anxiety Stress Scale.

^d^CSRI: Client Services Receipt Inventory.

^e^For families randomized to I-InTERACT-North.

#### Baseline Data

Chart extraction is performed by a research coordinator at baseline. Data collected include the following: CHD type, surgical history, weight and gestational age at birth, current and comorbid medical and mental health diagnoses, presence of pregnancy or delivery complications, seizures, vision problems, hearing problems, motor problems, and cerebral palsy.

For participants with neonatal magnetic resonance imaging data available, maximal total white matter injury (WMI) volume is documented and will be used for exploratory analyses in this study. WMI volume is calculated based on manual segmentation on the pre- and postoperative magnetic resonance imaging. WMI severity at each time point is categorized with a score of 0-3 (0: none; 1: minimal; 2: moderate; and 3: severe [6]).

Parents will also complete a background questionnaire, which includes questions assessing family history, demographic variables (ie, cultural identity, parents’ education, and employment status), and characteristics of the home environment (ie, number of people living in the home, languages spoken in the home).

### Primary Outcomes

Feasibility outcomes include program reach and adherence (ie, proportion of eligible families who enroll and complete the study) and program fidelity (assessed with a therapist checklist completed after every session).

Two primary outcomes are evaluated for the second objective: positive parenting skills and child behavior. Positive parenting skills are evaluated using the Dyadic Parent-Child Interaction Coding System (DPICS [34]). One member of a team of 3 coders, blind to condition, will observe a 5-minute recording of parent-child interactions and count the frequency of specific positive parenting skills (eg, labeled praise, behavioral descriptions, commands, questions, etc). In our team’s previous studies, interrater reliability coefficients were very high (>0.96 [28,29]). Throughout the RCT, 20% of all videos are double coded to prevent coder drift and coders meet regularly for consensus. In the case that reliability falls below α=.80, the team of coders will convene to reestablish consensus. The second primary outcome, child behavior, is measured with the parent-report ECBI [35]. The ECBI consists of 36 items describing various behavior concerns (eg, noncompliance, emotional regulation), each rated in terms of frequency from 1 (Never) to 7 (Always) as well as whether parents perceive it to be a problem for them (Yes/No). The ECBI yields an intensity score based on frequency of concerns and a problem score representing the number of concerns noted to be problems for the parents. Previous studies have demonstrated good convergent validity of the DPICS and the ECBI for assessing the impact of parenting skills on child behavior [34].

### Secondary Outcomes

Parent-reported secondary outcomes include quality of life, parent mental health, service receipt, and treatment acceptability. Quality of life is measured with the EQ-5D-5L ([36]), completed by the parent both for themselves and on behalf of their child. Parent mental health is measured with the Depression and Anxiety Stress Scale—Short Form [37]. Parents will also complete the Client Services Receipt Inventory [38] to report their use of mental health, community rehabilitation, neuropsychology, and educational psychology services. The Client Services Receipt Inventory also includes questions assessing the financial burden of mental health concerns (ie, cost of transportation to and from appointments, medications, alternative childcare, missed work, etc) Finally, for families randomized to I-InTERACT-North, parents will complete a Parent Acceptability Questionnaire at the end of treatment to report their understanding of the structure and purpose of the treatment.

### Sample Size and Power Analysis

Based on the size of the research registries available for recruitment and pilot estimates of consent rate (70%), we anticipate that 382 families will consent to participate in the RCT. Using pilot estimates of adherence rate (86%), we anticipate that 328, 282, and 244 families will complete the 3-month, 6-month, and 12-month follow-up measurements, respectively. Samples of 122 participants in each condition at 12 months postbaseline will provide >95% power to detect an effect size of 0.64, which is a medium to large effect size conservatively based on our pilot data. This sample size also achieves a power of 95% for subgroup analyses with subgroups with a split of 50%, which is consistent with or close to that of our pilot data.

### Planned Statistical Analysis

A mixed method approach will be taken with quantitative analyses conducted using SPSS Statistics (IBM Corp) [39] and qualitative analyses conducted using NVivo (Lumivero) [40].

#### Feasibility and Acceptability Analysis

Preliminary descriptive analyses will be used to assess the characteristics of families who participated in the trial, including each program step. Descriptive data will also be computed for adherence, program fidelity, costs, and maintenance. Parent reports of acceptability, adoption, and suggested adaptability of the program by families in the treatment condition, reported in the Parent Acceptability Questionnaire, will be examined qualitatively using cross-case thematic analyses [32,41]. Parent reports and the corresponding case analyses will be used to determine commonalities, differences, and inherent contextual properties that may explain these commonalities and differences across the cases [42]. The results of the case-specific analyses will be entered into a metamatrix to permit thematic analysis across cases. A final thematic table will be created to ensure a coherent, internally consistent, yet distinctive representation of the identified cross-case themes. The essence, scope, and content of the identified themes will be summarized in the final thematic table.

#### Primary Efficacy Analysis

Primary efficacy analysis will be conducted using an intent-to-treat approach. Generalized estimating equations for longitudinal count-level data having a Poisson distribution will be used to examine changes on the DPICS positive parenting composite. We will account for missing data with the Kenward-Roger degrees of freedom command and using a random intercept with the appropriate covariance structure to allow for unbalanced covariance matrices. The main effect of the program on positive parenting skills will be tested by linear contrast of adjusted DPICS mean change scores from baseline to 3 months between groups. We will also refit the regression model to include stratification variables (sex, baseline behavior intensity) and intervention step as moderators of the effect of treatment. The co–primary outcome of child behavior, as well as continuous secondary outcomes of quality of life and parent mental health, will be tested with repeated-measures analyses including condition, stratification variables (ie, sex assigned at birth and baseline child behavior severity), intervention step, and their interactions as fixed effects. Individual subjects as random effects will be adjusted to the data [43,44]. Sensitivity analyses will be carried out to replicate the analyses [29] from the initial adaptation of the I-InTERACT-North program into a stepped-care model for children with neurological risk, examining the preliminary efficacy in changes in positive parenting skills and child behavior based on intervention step. Finally, we will replicate the analyses conducted by Wade and colleagues [25] in the RCT of the original version of I-InTERACT for children with TBI, testing a moderated mediation model using regression-based process modeling to assess the indirect effects of treatment group on child behavior at 12-month follow-up through the change score for positive parenting.

## Results

**Figure 4:**
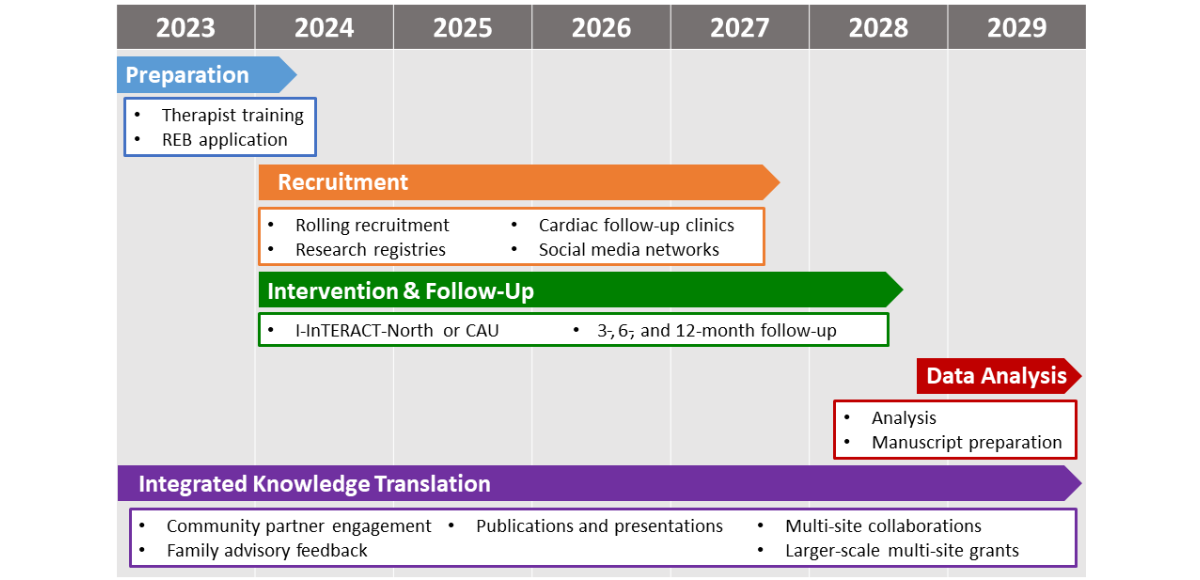
Study timeline. CAU: care as usual; I-InTERACT: Internet-based Interacting Together Everyday Recovery from Traumatic Brain Injury; REB: research ethics board.

The time period for this study is 5 years, having commenced alongside funding from the Canadian Institutes of Health Research (FRN186222) in February 2023 and research ethics board approval (Figure 4) in August 2023. Hiring and training of I-InTERACT-North therapists spanned April to August 2023, with a total of 13 active therapists as of April 2024. Recruitment began in February 2024. As of September 2024, 24 families have enrolled in the trial, and 55.0% (11/20) families randomized were assigned to the I-InTERACT-North condition. Therapy delivery began in March 2024 and is expected to continue until late 2028. Based on our pilot data and consent rate to date of 65%, we anticipate recruitment to continue through January 2028, with intervention and follow-up concluding in January 2029. An additional 8 months will be allocated toward data analyses and end-of-grant knowledge dissemination including publications in peer-reviewed journals and presentations at international conferences by August 2029.

Integrated knowledge translation will span the 5-year study period, including community partner engagement, family advisory feedback, preparation for knowledge dissemination, multisite collaborations, and preparation for larger-scale multisite grant applications nearing the study end date. To date, knowledge translation has included presentation and discussion with clinical teams in CHD clinics as well as parent and family advisors at SickKids. In February 2024, we consulted with a parent and family advisory council comprising parents and caregivers of children with CHD followed at The Hospital for Sick Children. Members endorsed the strong need for mental health supports, especially in middle childhood as mental health needs become apparent following stressful medical complexities in the early years. Parents also highlighted the lack of accessible mental health programming available, with one parent writing “When my daughter needed this 5 years ago nothing really existed at SickKids. We had to find support in the community which is also really challenging!” Similarly, nurses in the cardiac follow-up clinic at SickKids celebrated the acknowledgment of mental health needs for families and children with CHD and the availability of a free, adaptable, virtual program at SickKids.

## Discussion

### Principal Findings

This study protocol presents the rationale and details of a stepped-care web-based support program for parents of children with CHD and describes the trial that will be used to assess the feasibility, acceptability, and efficacy of I-InTERACT-North. To our knowledge, this is the only RCT to examine the efficacy of a virtual positive parenting program among families of children with CHD, contributing to the well-recognized gaps in mental health support in pediatric cardiology follow-up programs. As mortality from CHD has decreased and focus has shifted toward addressing developmental and mental health morbidities, the need for intervention programs has become a priority [45], and parents of children with CHD prioritize mental health needs among the most important factors in optimizing their child’s and family’s well-being [7,8,14,46]. However, existing CHD literature regarding intervention supports has concentrated primarily on early developmental services, psychopharmacological treatment, and the need for academic supports, with limited psychosocial supports available [47]. Thus, the critical need for accessible, evidence-based, and personalized mental health supports, taken together with evidence supporting positive parenting as a modifiable determinant of health and the relationship between psychosocial support and psychological distress among families of children with CHD [48], underlines the need for this trial.

This trial aligns with the Cardiac Neurodevelopmental Outcome Collaborative’s clinically informed research agenda to “adapt existing interventions for individuals with CHD” to advance this field of research and elucidate the impact of psychosocial interventions in CHD populations [49]. The hybrid type 1 design was selected purposefully to simultaneously test both intervention efficacy and implementation outcomes, thereby shortening the time needed to study both [50]. The hybrid design is preferred when evidence exists in support of the intervention (as is the case with I-InTERACT-North) and when there is momentum for implementation [50,51]. Examining stepped care also increases potential for improved service delivery and access for at-risk and diverse families traditionally underrepresented in mental health research.

### Comparison With Prior Works

Despite significant empirical support for parent management training and behavioral family interventions for children and adolescents with developmental and behavioral challenges [52-55], few studies have specifically targeted CHD populations. The work of Christopher McCusker and his team at The Royal Belfast Hospital for Sick Children, Northern Ireland, has pioneered this work across distinct age groups, with 3 Congenital Heart Disease Intervention Programs (CHIP; ie, CHIP-Infant [56], CHIP-School [57], and CHIP-Family [58]). CHIP-Infant is a 6-session early intervention program that supports infant mental development, feeding, and maternal mental health, with improvements demonstrated 6 months post intervention [56]. CHIP-School and CHIP-Family are designed for school-aged children aged 4-5 and 5-8 years, respectively [57,58]. Both involve single-day parent workshops and follow-up sessions focusing on problem prevention therapy, psychoeducation, and parenting skills. CHIP-School also includes parent observation of their child safely engaging in exercise to demonstrate the child’s ability to tolerate physical activity. CHIP-Family offers child-specific cognitive-behavior therapy strategies, light exercise, and activities that promote self-esteem, emotion regulation, and problem-solving. Participation in CHIP-School was associated with improvements in maternal mental health, family functioning, and school attendance 10 months post participation but no significant improvements in child emotional and behavioral concerns. Notably, the CHIP-School study excluded children with neurodevelopmental disorders, limiting generalizability. Similarly, participation in CHIP-Family yielded no significant differences at 6-month follow-up relative to a control group in child emotional and behavioral concerns.

Taken together, the CHIP programs provide preliminary support that parent-centered interventions yield valuable initial gains with respect to parental mental health and family functioning in CHD [56-58]; however, the CHIP programs primarily target parental adjustment to CHD and have a limited focus on positive parenting skills and the parent-child relationship. Similarly, other programs have also targeted the intensive early stress in infancy following CHD diagnosis or treatment for parents. Among parents of children with CHD, Li et al [59] demonstrated significant reduction in parental distress and heightened parental hope for those who underwent a solution-focused brief therapy for parental distress. Likewise, Fleck et al [60] are conducting an RCT to evaluate the efficacy of the REACH intervention for families with infants recovering from surgery for complex CHD. REACH is a home-monitoring program that combines technology and advance practice nurse monitoring to decrease parental stress and improve parental quality of life and infant stability. Although parent-focused interventions may help support early parental coping and adjustment to CHD, they may not extend to later parent-child relationship support. Other psychological intervention research with CHD populations has focused on therapy directed exclusively to children and adolescents, with little to no parental involvement and parent support [61,62].

In reviewing the landscape of the literature to date, the need for evidenced-based and personalized mental health supports for parenting and childhood behavioral problems in CHD is highlighted. Through the proposed RCT, we hope to illustrate the efficacy, feasibility, and acceptability of a novel web-based stepped-care program in improving positive parenting skills and child behavior among families of children with CHD. This trial represents the first examination of a positive parenting intervention program designed with and for families of children with CHD. Embedded within a well-established cardiac follow-up program at The Hospital for Sick Children, we have the unique opportunity to leverage a large, prospective cohort of children with a history of CHD. These children and families have longitudinal neurodevelopmental and neuroimaging data collected since birth. If the effects of the intervention are positive, as we hypothesize given previous pilot trials, this study can contribute toward widespread implementation and expansion. This implementation can span nationally and globally across various CHD cohorts, while leading the way in elucidating key mechanisms of change in treating emotional and behavioral challenges in children with CHD. As such, this study can inform the application of this program to support families of children with other early medical conditions.

### Limitations

Several limitations will be taken into consideration with respect to this clinical trial. This is a single-site study that reflects findings specific to The Hospital for Sick Children, the Toronto, Ontario, context, and a public health care system. Recruitment and intervention materials are currently available only in English, thus limiting access and generalizability to non–English-speaking families. However, several therapists are bilingual or multilingual (English and French) and interpreter services are available for recruitment, informed consent discussions, and web-based coaching sessions. Likewise, we provide families in need with tablets and web-based sticks as necessary to ensure that web-based coverage status does not pose a barrier to participation. Although the stepped-care treatment model offers increased customization and precision in our intervention approach to optimally address families’ needs, it may be challenging to elucidate differences in outcomes between those who receive exclusively an introductory appointment relative to the CAU group. It may also be difficult to control the effects of concurrent intervention services (eg, psychotherapy, rehabilitation) in evaluating the efficacy of I-InTERACT-North; however, service receipt will be explored as a secondary objective to ascertain valuable insight into additional mental health supports sought by families.

### Conclusions

There is a lack of accessible, evidence-based behavioral interventions available for CHD children and families. This web-based, stepped-care mental health program was designed to support family well-being and reduce behavioral concerns through positive parenting and psychoeducation regarding CHD. In implementing this research protocol, we expect to provide evidence for the program’s effectiveness in increasing positive parenting skills and decreasing child behavior problems in families of children with CHD, while also demonstrating high parent-reported acceptability. This study can provide evidence to support future widespread clinical implementation and expansion. Likewise, our findings can help elucidate mechanisms of change in treating emotional and behavioral challenges in children with CHD. Overall, the clinical and research implications derived from this study can support the mental health and well-being of children and families who experience CHD and advance the field of psychological interventions in this population more broadly.

## Appendix

Multimedia Appendix 1 Peer-review report by Project Grant/Subvention Projet - Committee / Comité: Social & Developmental Aspects of Children's & Youth's Health/Aspects sociaux et développementaux de la santé de l'enfant et de l'adolescent - Canadian Institutes of Health Research/Instituts de recherche en santé du Canada (CIHR/IRSC) (Canada).

## Abbreviations

CAU: care as usual
CHD: congenital heart disease
CHIP: Congenital Heart Disease Intervention Programs
DPICS: Dyadic Parent-child Interaction Coding System
ECBI: Eyberg Child Behavior Inventory
I-InTERACT: Internet-based Interacting Together Everyday Recovery from Traumatic Brain Injury
RCT: randomized controlled trial
REDCap: Research Electronic Data Capture
TBI: traumatic brain injury
WMI: white matter injury

## Data Availability

The data sets generated during or analyzed during this study will be made available upon study completion by reasonable request to the corresponding author.
