# Supplementary material for: Stepped-Care Web-Based Parent Support Following Congenital Heart Disease: Protocol for a Randomized Controlled Trial
Source: JMIR Res Protoc. 2024 Oct 4;13:e64216. doi: 10.2196/64216 (PMC11489793; doi:10.2196/64216)
Supplement: Multimedia Appendix 1 [file resprot_v13i1e64216_app1.pdf]

**Review Type / Type d'évaluation:**	Reviewer 1 / Évaluateur 1
**Name of Applicant / Nom du chercheur:**	Williams, Tricia
**Application No. / Numéro de demande:**	485988
**Agency / Agence:**	CIHR/IRSC
**Competition / Concours:**	Project Grant/Subvention Projet
**Committee / Comité:**	Social & Developmental Aspects of Children's & Youth's Health/Aspects sociaux et développementaux de la santé de l'enfant et de l'adolescent
**Title / Titre:**	Stepped-Care Online Parent Training following Congenital Heart Disease: A Randomized Control Trial

Adjudication Criteria/Critères de sélection

Initial Score/Cote Initiale: 4.3

Top/Bottom Selection/Groupe supérieur/inférieur

- (●) **Top/Groupe supérieur**
- ( ) **Bottom/Groupe inférieur**

**Review Type / Type d'évaluation:**	Reviewer 1 / Évaluateur 1
**Name of Applicant / Nom du chercheur:**	Williams, Tricia
**Application No. / Numéro de demande:**	485988
**Agency / Agence:**	CIHR/IRSC
**Competition / Concours:**	Project Grant/Subvention Projet
**Committee / Comité:**	Social & Developmental Aspects of Children's & Youth's Health/Aspects sociaux et développementaux de la santé de l'enfant et de l'adolescent
**Title / Titre:**	Stepped-Care Online Parent Training following Congenital Heart Disease: A Randomized Control Trial

Summary of Application/Résumé de la demande:

This is the second resubmission of an earlier grant proposal.

Innovations in medical care have resulted in dramatic improvement in survival of children born with congenital heart disease (CHD). Yet, these children continue to face significantly more learning, socioemotional, and behavioural challenges compared to their term-born healthy peers. These challenges place enormous burdens on the family, education, and healthcare systems, yet with longstanding gaps in mental health service. Widely used interventions for early socioemotional and behavioural challenges promote positive parenting skills that lead to improved child behaviour.

This proposal focuses on I-InTERACT-North which is a virtual parenting program that has been tested in 2 pilot trial among a transdiagnostic group of children which included children with CHD. They have gone on to develop a stepped care program which will focus on optimizing treatment length and intensity based on family needs.

The research group will use a Hybrid Type 1 efficacy-implementation design with the Primary Aim to establish evidence of efficacy for I-InTERACT-North; and a Secondary Aim to gather feasibility and acceptability indicators (e.g., adherence, fidelity, costs, acceptability) of the stepped-care program from key stakeholders to inform a future multi-site trial. The purpose of using a hybrid design is combining goals into one study with the hopes of reducing overall time to expedite a program of research to practice. A third aim will be to examine the association between neonatal white matter injury severity on treatment and implementation outcomes; this is an exploratory aim.

The stepped program contains 3 tier-stepped of increasing therapeutic support. Step 1 is a podcast whereas step 2 and 3 is a combination of website modules with video conferencing coaching sessions. All session will be recorded. Outcomes measures will be made on observed parenting skills and there will be no additional questionnaires. Controls will be care as usual which is not intensive support.

Participants will be randomized but balance by child sex as assigned at birth, severity of brain injury and baseline behaviour concern intensity . looking at enrolling where children are between 3-9 years of age.

Burden on family seems reasonable: step 1- 30 min podcast; step 2- 2 I hour sessions; and step 3 are 5-1 hour sessions done weekly. Follow-up questionnaires will be done at 3, 6, 12, and 18 months post intervention for both groups.

Primary outcome will be “positive parenting skills” through blinded coding of the recorded interactions plus now a co-primary outcome which is a child behaviour checklist. Secondary outcomes will look at quality of life measures as well as measure of parental health.

Plan to enroll 328 families which will enable to maintain an adequate number of participants at the defined follow-up visits.

Aim 2 looks at implementation outcome measures.

**Review Type / Type d'évaluation:**	Reviewer 1 / Évaluateur 1
**Name of Applicant / Nom du chercheur:**	Williams, Tricia
**Application No. / Numéro de demande:**	485988
**Agency / Agence:**	CIHR/IRSC
**Competition / Concours:**	Project Grant/Subvention Projet
**Committee / Comité:**	Social & Developmental Aspects of Children's & Youth's Health/Aspects sociaux et développementaux de la santé de l'enfant et de l'adolescent
**Title / Titre:**	Stepped-Care Online Parent Training following Congenital Heart Disease: A Randomized Control Trial

Strengths and Weaknesses/Forces et faiblesses:

This is an interesting project and a solid team to carry off this project. The investigator is a mid-career investigator who has been very productive and has done a number of project along a similar vein. She appears capable of leading the project and has solid support behind her.

The research group has responded appropriately to the reviewers. A major criticism of the two previous submissions was the population to be studied. The previous submissions included not only children with CHD but also preterm infants. For this submission they agreed in principle and now designed the trial to only include CHD children which is cleaner.

It is not clear how the control group will be recorded to assess the outcome measures in this group.

There appears to be a lot of measures and the timeline of how these measures will be captured at each of the follow-up visits.is not clear.

Outcome measures were very convoluted and I lost sight of what was the primary outcome for the trial and then they started to talk of other outcome measures. It appears that there will be a primary outcome measures at each follow-up visit. This contributed to clarity issues related to how the grant was written but did not take away the value of the project. The project may benefit from clarifying the primary outcomes for the trial overall like it was outlined in the summary and then clarify the other outcomes that is being referred to. A table outlining what was going to be done at each time point and what was going to be analyzed and considered would be helpful.

**Review Type / Type d'évaluation:**	Reviewer 1 / Évaluateur 1
**Name of Applicant / Nom du chercheur:**	Williams, Tricia
**Application No. / Numéro de demande:**	485988
**Agency / Agence:**	CIHR/IRSC
**Competition / Concours:**	Project Grant/Subvention Projet
**Committee / Comité:**	Social & Developmental Aspects of Children's & Youth's Health/Aspects sociaux et développementaux de la santé de l'enfant et de l'adolescent
**Title / Titre:**	Stepped-Care Online Parent Training following Congenital Heart Disease: A Randomized Control Trial

Budget Recommendation/Recommandation budgétaire:

Largely reasonable.

Please indicate your appraisal of the integration of sex as a biological variable as a strength, weakness, or not applicable to the proposal./Prière de sélectionner une option pour donner votre évaluation de l'intégration du sexe comme variable biologique en tant que point fort ou point faible de la proposition, ou en tant qu'élément non applicable à la proposition.

- (●) **Strength/Point fort**
- ( ) **Weakness/Point faible**
- ( ) **Not applicable/Non applicable**

Please indicate your appraisal of the integration of gender as a socio-cultural determinant of health as a strength, weakness, or not applicable to the proposal./Prière de sélectionner une option pour donner votre évaluation de l'intégration du genre comme déterminant socioculturel de la santé en tant que point fort ou point faible de la proposition, ou en tant qu'élément non applicable à la proposition.

- (●) **Strength/Point fort**
- ( ) **Weakness/Point faible**
- ( ) **Not applicable/Non applicable**

**Review Type / Type d'évaluation:**	Reviewer 1 / Évaluateur 1
**Name of Applicant / Nom du chercheur:**	Williams, Tricia
**Application No. / Numéro de demande:**	485988
**Agency / Agence:**	CIHR/IRSC
**Competition / Concours:**	Project Grant/Subvention Projet
**Committee / Comité:**	Social & Developmental Aspects of Children's & Youth's Health/Aspects sociaux et développementaux de la santé de l'enfant et de l'adolescent
**Title / Titre:**	Stepped-Care Online Parent Training following Congenital Heart Disease: A Randomized Control Trial

Sex and/or Gender Considerations/Notions de sexe et/ou de genre:

**Review Type / Type d'évaluation:**	Reviewer 2 / Évaluateur 2
**Name of Applicant / Nom du chercheur:**	Williams, Tricia
**Application No. / Numéro de demande:**	485988
**Agency / Agence:**	CIHR/IRSC
**Competition / Concours:**	Project Grant/Subvention Projet
**Committee / Comité:**	Social & Developmental Aspects of Children's & Youth's Health/Aspects sociaux et développementaux de la santé de l'enfant et de l'adolescent
**Title / Titre:**	Stepped-Care Online Parent Training following Congenital Heart Disease: A Randomized Control Trial

Adjudication Criteria/Critères de sélection

Initial Score/Cote Initiale: 4.5

Top/Bottom Selection/Groupe supérieur/inférieur

- (●) **Top/Groupe supérieur**
- ( ) **Bottom/Groupe inférieur**

**Review Type / Type d'évaluation:**	Reviewer 2 / Évaluateur 2
**Name of Applicant / Nom du chercheur:**	Williams, Tricia
**Application No. / Numéro de demande:**	485988
**Agency / Agence:**	CIHR/IRSC
**Competition / Concours:**	Project Grant/Subvention Projet
**Committee / Comité:**	Social & Developmental Aspects of Children's & Youth's Health/Aspects sociaux et développementaux de la santé de l'enfant et de l'adolescent
**Title / Titre:**	Stepped-Care Online Parent Training following Congenital Heart Disease: A Randomized Control Trial

Summary of Application/Résumé de la demande:

Stepped-Care Online Parent Training following Congenital Heart Disease: A Randomized Control Trial

The investigators propose to test the effectiveness of I-nTERACT-North stepped care program on positive parenting skills and child behaviour for families of children with a history of congenital heart disease (CHD). A secondary purpose is to examine the feasibility (e.g., adherence, cost, fidelity) and acceptability among parents, medical providers and therapists. A third aim is to explore whether white matter injury severity is associated with treatment outcomes and feasibility.

**Review Type / Type d'évaluation:**	Reviewer 2 / Évaluateur 2
**Name of Applicant / Nom du chercheur:**	Williams, Tricia
**Application No. / Numéro de demande:**	485988
**Agency / Agence:**	CIHR/IRSC
**Competition / Concours:**	Project Grant/Subvention Projet
**Committee / Comité:**	Social & Developmental Aspects of Children's & Youth's Health/Aspects sociaux et développementaux de la santé de l'enfant et de l'adolescent
**Title / Titre:**	Stepped-Care Online Parent Training following Congenital Heart Disease: A Randomized Control Trial

Strengths and Weaknesses/Forces et faiblesses:

There are many strengths of this application. The topic is highly relevant. The investigators have successfully conducted preliminary research (pilot) that informs the present application and the quality of the methodological procedures were well-considered. Investigators also completed (and published) a systematic review to inform the proposed study.

The intervention is well-designed and tailored based on family needs to provide support from the least to most intensive needs.

Strong research design: single-blind, single-site, RCT, intervention vs care as usual

Based on their past research, the investigators could estimate participation and retention and knowing the size of the pool from which participants will be recruited, estimates may be realistic. A sample size calculation was performed for the specific outcome. The sample size estimate required for a successful project also aligns with the size of the pool of participants and the estimated participation rates.

An appropriate method of randomization completed by research coordinator external or independent from the team is proposed.

There were a few areas to strengthen. The plan includes a monitoring of sought or used adjunct therapies or support though it might be stronger to regularly ask participants to report the use of these therapies and potentially control for their use in the analyses. In the analytic plan (page 10 of 10) it is stated that for subgroup analyses, mother-completed data will be analyzed followed by father-completed data. Could there be other configurations of parents/guardians/caregivers, sex/gender diversity, and so forth?

Outcomes were well considered – it could have been clarified that both outcomes for both intervention and control groups were being captured in the same way. Including the section on Setting in the Methods would be helpful (see CONSORT https://www.consort-statement.org/).

**Review Type / Type d'évaluation:**	Reviewer 2 / Évaluateur 2
**Name of Applicant / Nom du chercheur:**	Williams, Tricia
**Application No. / Numéro de demande:**	485988
**Agency / Agence:**	CIHR/IRSC
**Competition / Concours:**	Project Grant/Subvention Projet
**Committee / Comité:**	Social & Developmental Aspects of Children's & Youth's Health/Aspects sociaux et développementaux de la santé de l'enfant et de l'adolescent
**Title / Titre:**	Stepped-Care Online Parent Training following Congenital Heart Disease: A Randomized Control Trial

Budget Recommendation/Recommandation budgétaire:

None

**Review Type / Type d'évaluation:**	Reviewer 2 / Évaluateur 2
**Name of Applicant / Nom du chercheur:**	Williams, Tricia
**Application No. / Numéro de demande:**	485988
**Agency / Agence:**	CIHR/IRSC
**Competition / Concours:**	Project Grant/Subvention Projet
**Committee / Comité:**	Social & Developmental Aspects of Children's & Youth's Health/Aspects sociaux et développementaux de la santé de l'enfant et de l'adolescent
**Title / Titre:**	Stepped-Care Online Parent Training following Congenital Heart Disease: A Randomized Control Trial

Please indicate your appraisal of the integration of sex as a biological variable as a strength, weakness, or not applicable to the proposal./Prière de sélectionner une option pour donner votre évaluation de l'intégration du sexe comme variable biologique en tant que point fort ou point faible de la proposition, ou en tant qu'élément non applicable à la proposition.

- (●) **Strength/Point fort**
- ( ) **Weakness/Point faible**
- ( ) **Not applicable/Non applicable**

Please indicate your appraisal of the integration of gender as a socio-cultural determinant of health as a strength, weakness, or not applicable to the proposal./Prière de sélectionner une option pour donner votre évaluation de l'intégration du genre comme déterminant socioculturel de la santé en tant que point fort ou point faible de la proposition, ou en tant qu'élément non applicable à la proposition.

- (●) **Strength/Point fort**
- ( ) **Weakness/Point faible**
- ( ) **Not applicable/Non applicable**

**Review Type / Type d'évaluation:**	Reviewer 2 / Évaluateur 2
**Name of Applicant / Nom du chercheur:**	Williams, Tricia
**Application No. / Numéro de demande:**	485988
**Agency / Agence:**	CIHR/IRSC
**Competition / Concours:**	Project Grant/Subvention Projet
**Committee / Comité:**	Social & Developmental Aspects of Children's & Youth's Health/Aspects sociaux et développementaux de la santé de l'enfant et de l'adolescent
**Title / Titre:**	Stepped-Care Online Parent Training following Congenital Heart Disease: A Randomized Control Trial

Sex and/or Gender Considerations/Notions de sexe et/ou de genre:

Sex and gender were well- considered though explicit description in the analysis section could enhance the application.

**Review Type / Type d'évaluation:**	Reviewer 3 / Évaluateur 3
**Name of Applicant / Nom du chercheur:**	Williams, Tricia
**Application No. / Numéro de demande:**	485988
**Agency / Agence:**	CIHR/IRSC
**Competition / Concours:**	Project Grant/Subvention Projet
**Committee / Comité:**	Social & Developmental Aspects of Children's & Youth's Health/Aspects sociaux et développementaux de la santé de l'enfant et de l'adolescent
**Title / Titre:**	Stepped-Care Online Parent Training following Congenital Heart Disease: A Randomized Control Trial

Adjudication Criteria/Critères de sélection

Initial Score/Cote Initiale: 4.7

Top/Bottom Selection/Groupe supérieur/inférieur

- (●) **Top/Groupe supérieur**
- ( ) **Bottom/Groupe inférieur**

**Review Type / Type d'évaluation:**	Reviewer 3 / Évaluateur 3
**Name of Applicant / Nom du chercheur:**	Williams, Tricia
**Application No. / Numéro de demande:**	485988
**Agency / Agence:**	CIHR/IRSC
**Competition / Concours:**	Project Grant/Subvention Projet
**Committee / Comité:**	Social & Developmental Aspects of Children's & Youth's Health/Aspects sociaux et développementaux de la santé de l'enfant et de l'adolescent
**Title / Titre:**	Stepped-Care Online Parent Training following Congenital Heart Disease: A Randomized Control Trial

Summary of Application/Résumé de la demande:

This proposal relates to parenting skills and child behaviour in children with congenital heart disease. This is a 3rd or 4th resubmission. The investigators did a good job reviewing to the comments in this round.

The objectives are to:

1. determine the efficacy of the I-InTERACT-North stepped-care program compared to care as usual in improving positive parenting skills (primary outcome) and reducing child emotional and behavioural problems (co-primary outcome) among CHD children.
2. examine feasibility and acceptability of the stepped-care program among stakeholders (parents, therapists, medical providers).
3. examine the association between neonatal white matter injury severity on treatment and implementation outcomes.

The investigators are carrying out an RCT among 575 children with CHD. They are examining the effect of a stepped-care intervention (I Interact-North) to improve parenting skills and child behavior

**Review Type / Type d'évaluation:**	Reviewer 3 / Évaluateur 3
**Name of Applicant / Nom du chercheur:**	Williams, Tricia
**Application No. / Numéro de demande:**	485988
**Agency / Agence:**	CIHR/IRSC
**Competition / Concours:**	Project Grant/Subvention Projet
**Committee / Comité:**	Social & Developmental Aspects of Children's & Youth's Health/Aspects sociaux et développementaux de la santé de l'enfant et de l'adolescent
**Title / Titre:**	Stepped-Care Online Parent Training following Congenital Heart Disease: A Randomized Control Trial

Strengths and Weaknesses/Forces et faiblesses:

Strengths

-addresses parenting skills in a population at need (a population at risk of brain injury)

-provides new information regarding the neurodevelopmental and psychosocial impact of congenital and neonatal conditions including CHD

-have stepped-care pilot data (described below) that demonstrates the program’s preliminary success in improving positive parenting

-excellent KT plan

-RCT plan is well described; there will be a podcast and coaching sessions

-all measures are well detailed and justified

-clear power calculations

-This study is feasible and it is clear that it will achieve its objectives

**Review Type / Type d'évaluation:**	Reviewer 3 / Évaluateur 3
**Name of Applicant / Nom du chercheur:**	Williams, Tricia
**Application No. / Numéro de demande:**	485988
**Agency / Agence:**	CIHR/IRSC
**Competition / Concours:**	Project Grant/Subvention Projet
**Committee / Comité:**	Social & Developmental Aspects of Children's & Youth's Health/Aspects sociaux et développementaux de la santé de l'enfant et de l'adolescent
**Title / Titre:**	Stepped-Care Online Parent Training following Congenital Heart Disease: A Randomized Control Trial

Budget Recommendation/Recommandation budgétaire:

1 million requested, seems ok

**Review Type / Type d'évaluation:**	Reviewer 3 / Évaluateur 3
**Name of Applicant / Nom du chercheur:**	Williams, Tricia
**Application No. / Numéro de demande:**	485988
**Agency / Agence:**	CIHR/IRSC
**Competition / Concours:**	Project Grant/Subvention Projet
**Committee / Comité:**	Social & Developmental Aspects of Children's & Youth's Health/Aspects sociaux et développementaux de la santé de l'enfant et de l'adolescent
**Title / Titre:**	Stepped-Care Online Parent Training following Congenital Heart Disease: A Randomized Control Trial

Please indicate your appraisal of the integration of sex as a biological variable as a strength, weakness, or not applicable to the proposal./Prière de sélectionner une option pour donner votre évaluation de l'intégration du sexe comme variable biologique en tant que point fort ou point faible de la proposition, ou en tant qu'élément non applicable à la proposition.

- ◉ **Strength/Point fort**
- ◯ **Weakness/Point faible**
- ◯ **Not applicable/Non applicable**

Please indicate your appraisal of the integration of gender as a socio-cultural determinant of health as a strength, weakness, or not applicable to the proposal./Prière de sélectionner une option pour donner votre évaluation de l'intégration du genre comme déterminant socioculturel de la santé en tant que point fort ou point faible de la proposition, ou en tant qu'élément non applicable à la proposition.

- ◉ **Strength/Point fort**
- ◯ **Weakness/Point faible**
- ◯ **Not applicable/Non applicable**

**Review Type / Type d'évaluation:**	Reviewer 3 / Évaluateur 3
**Name of Applicant / Nom du chercheur:**	Williams, Tricia
**Application No. / Numéro de demande:**	485988
**Agency / Agence:**	CIHR/IRSC
**Competition / Concours:**	Project Grant/Subvention Projet
**Committee / Comité:**	Social & Developmental Aspects of Children's & Youth's Health/Aspects sociaux et développementaux de la santé de l'enfant et de l'adolescent
**Title / Titre:**	Stepped-Care Online Parent Training following Congenital Heart Disease: A Randomized Control Trial

Sex and/or Gender Considerations/Notions de sexe et/ou de genre:

Parent gender is binary – this is one aspect that could be improved. Child sex will be considered.
